# Non-coding RNA in cartilage regeneration: regulatory mechanism and therapeutic strategies

**DOI:** 10.3389/fbioe.2025.1522303

**Published:** 2025-03-26

**Authors:** Mengnan Wen, Xueqiang Guo, Jingdi Zhang, Yunian Li, Jixiang Li, Zhenlin Fan, Wenjie Ren

**Affiliations:** ^1^ Institutes of Health Central Plain, The Third Affiliated Hospital of Xinxiang Medical University, Clinical Medical Center of Tissue Engineering and Regeneration, Xinxiang Medical University, Xinxiang, China; ^2^ Henan Key Laboratory for Medical Tissue Regeneration, School of Basic Medical Sciences, Xinxiang Medical University, Xinxiang, China; ^3^ Junji College of Xinxiang Medical University, Xinxiang Medical University, Xinxiang, China

**Keywords:** cartilage regeneration, non-coding RNA, gene therapy, tissue engineering and regenerative medicine, OA

## Abstract

The pathogenesis of cartilage injury and degeneration is exceptionally complex. In addition to being associated with osteoarthritis and trauma, factors such as age, gender, obesity, inflammation, and apoptosis of chondrocytes are also considered significant influencing factors. Due to the lack of direct blood supply, lymphatic circulation, and neural innervation, coupled with low metabolic activity, the self-repair capability of cartilage after injury is extremely limited, making its treatment quite challenging. Recent research indicated that ncRNA, a class of RNA transcribed from the genome that does not encode proteins, played a crucial regulatory role in various disease processes. Particularly noteworthy is its positive regulatory role in cartilage regeneration, achieved through the modulation of the inflammatory microenvironment, promotion of chondrocyte proliferation, inhibition of chondrocyte degradation, and facilitation of the recruitment and differentiation of bone marrow mesenchymal stem cells into chondrocytes. In the earlier phase, we conducted a review and outlook on therapeutic strategies for the regeneration of articular cartilage injuries. This article specifically focuses on summarizing the regulatory roles and research advancements of ncRNA in cartilage regeneration, as well as its contributions to the clinical application of gene therapy for cartilage defects.

## 1 Introduction

**FIGURE 1:**
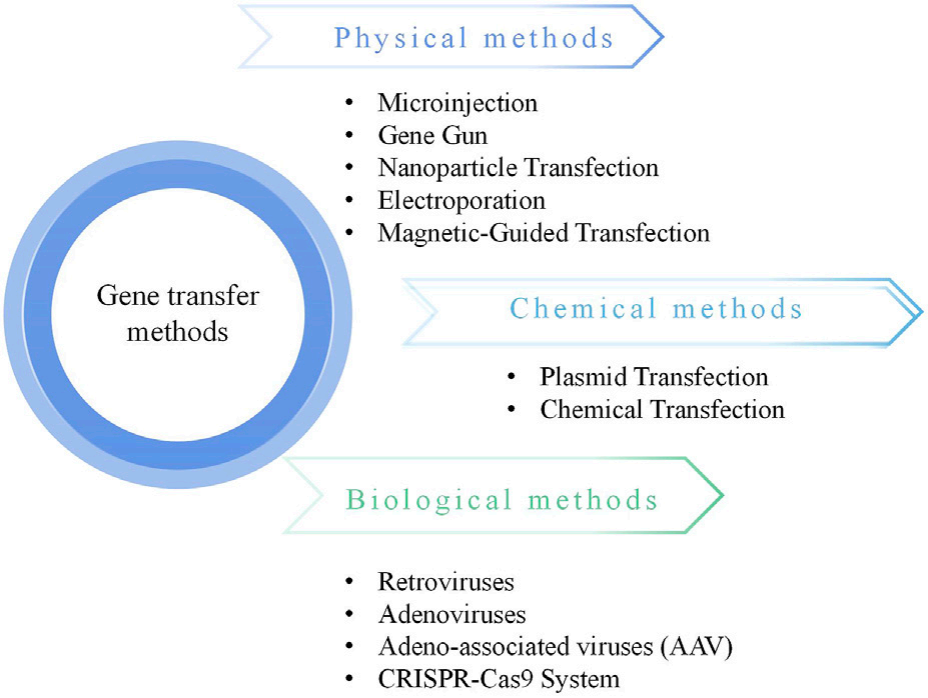
Gene transfer methods.

Cartilage injury and degradation represent common progressive conditions, with osteoarthritis (OA) being the most prevalent degenerative disease of synovial joints, leading to chronic disability due to pain and associated joint dysfunction (Hunter and Bierma-Zeinstra, 2019). Currently, there is no effective treatment to halt the progression of OA, and many patients ultimately resort to surgical joint replacement. Cartilage destruction lies at the core of the pathogenesis of osteoarthritic conditions, mediated by matrix-degrading enzymes (Shen et al., 2021a). Due to limited nutrient supply, damaged hyaline cartilage lacks the ability to self-repair, rendering OA challenging to treat. Gene therapy, defined as “the administration of genetic material to regulate its effects by transcription and/or translation and/or integration into the host genome, delivered in the form of nucleic acids, viruses, or genetically engineered microorganisms”, has emerged as a powerful tool imperative for correcting diseases at the genetic level (Sayed et al., 2022). In recent years, an increasing number of gene therapy products have gained clinical approval, making gene therapy for diseases a tangible possibility (Figure 1).

Non-coding RNA (ncRNA) is a widely present RNA subset in eukaryotes that does not encode proteins. Studies have revealed the regulatory mechanisms of ncRNA in various diseases, and it holds clinical potential for promoting cartilage regeneration. Growing evidence indicates an intimate correlation between abnormal ncRNA expression and the development of OA (Izda et al., 2021). There are also increasing studies on the *in vivo* treatment of ncRNA. For example, researcher found that lipid nanoparticle (LNPs) loaded with diverse mRNA cargos can effectively reach hematopoietic stem cells (HSCs) within the mouse bone marrow niche through a single systemic injection. The delivery systems hold the potential to bridge the gap between decades of collaborative genetic and biomedical research and the treatment of a broad spectrum of human diseases (Breda et al., 2023).

## 2 Non-coding RNA

**FIGURE 2:**
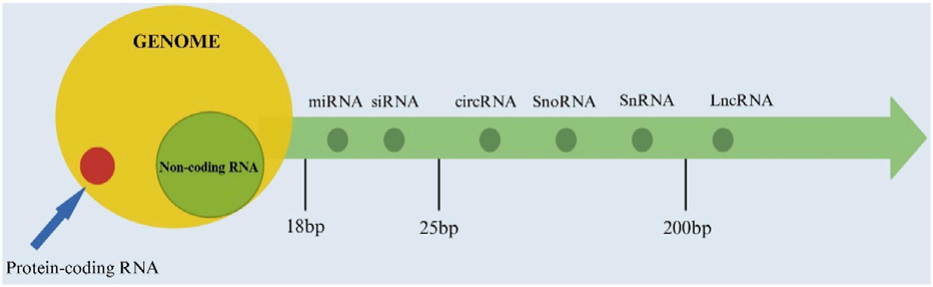
The location of non-coding RNA.

NcRNA refers to a class of RNA molecules transcribed from the genome that do not encode proteins but play crucial regulatory roles in various biological processes, including gene expression, chromatin remodeling, and cellular signaling (Table 1). Based on their length, ncRNA can be divided into three categories: less than 50 nt, including microRNA (miRNA), small interfering RNA (siRNA), etc., 50 to 200 nt, including ribosomal RNA (rRNA), etc., greater than 200 nt, representing long noncoding RNA (lncRNA), which encompasses lncRNA with messenger RNA (mRNA)-like features and lncRNA without a poly A tail, and a distinct class with a closed circular structure known as circular RNA (circRNA). In addition, there are also some ncRNA with lengths ranging between the aforementioned ones, such as small nucleolar RNA (snoRNA) and small nuclear RNA (snRNA) from 50 to 300, etc. (Deogharia and Gurha, 2022). The position of the ncRNA is shown in Figure 2.

**TABLE 1:** The classification of ncRNA.

Types	Length	Location	Main functions
CircRNA	Variable	Cytoplasm or EVs	1. Regulates the expression and splicing of parent genes; 2. Forms complexes with proteins to execute biological functions; 3. Modulates gene expression as miRNA sponge
MiRNA	18–25 nt	Intronic regions or intergenic	Pairs with target gene mRNA to guide the silencing complex, leading to mRNA degradation or translation inhibition, thereby regulating gene expression
LncRNA	>200 nt	Nucleus or cytoplasm	1. Genomic imprinting; 2. Transcriptional interference; 3. Transcriptional activation; 4. Chromatin modification; 5. Chromosome silencing
SnoRNA	50–250 nt	Nucleolus	Involved in various cellular processes, known for metabolic stability
SnRNA	50–200 nt	Nucleus	Forms small nuclear ribonucleoproteins (snRNPs) with proteins, plays a crucial role in processing RNA precursors, particularly in intron excision
SiRNA	∼22 nt	Cytoplasm	Processed by Dicer, a product of RNA interference (RNAi), initially designed to inhibit transposon activity and viral infections

## 3 Cartilage regeneration

### 3.1 The composition of cartilage

**FIGURE 3:**
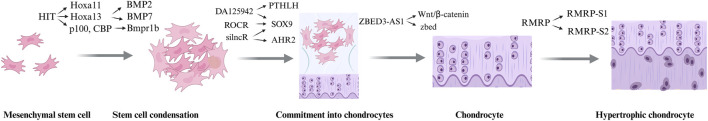
The process of cartilage development.

The tissue known as cartilage is composed of chondrocytes and extracellular matrix (ECM). Despite its seemingly simple structure, which primarily consists of a single cell type (chondrocytes) and an ECM dominated by water, collagen, and proteoglycans (Hollander et al., 2010), cartilage development is a highly intricate and dynamic process. This process involves five distinct yet interconnected stages (Figure 3): (1) the initial commitment of mesenchymal stem cells (MSCs) to the chondrogenic lineage, (2) their transformation into chondrocytes, (3) the subsequent differentiation of chondrocytes, (4) chondrocyte hypertrophy, and (5) the final stages of cartilage matrix calcification and degradation (Zhu et al., 2019). Each stage is tightly regulated by a complex interplay of signaling pathways, transcription factors, and extracellular cues, ensuring the proper formation and maintenance of cartilage tissue. This multi-step process highlights the sophisticated biological mechanisms underlying cartilage development, far beyond a simple linear progression.

It is essential to differentiate between terminal chondrocytes derived from MSCs, which are involved in endochondral ossification and bone formation, and articular chondrocytes, which form the stable cartilage at the surface of joints. Articular cartilage is characterized by its avascular nature, low cellularity, and limited regenerative capacity. Once the superficial layer of articular cartilage is damaged, the access to chondroprogenitors in the zone of Ranvier, located at the cartilage-bone junction, is severely compromised. This zone contains a population of chondroprogenitor cells that are critical for cartilage repair. However, their limited accessibility and the harsh inflammatory microenvironment in OA further hinder the regeneration of articular cartilage.

### 3.2 The classification of cartilage

Hyaline cartilage, elastic cartilage, and fibrocartilage are three distinct types of cartilage, each serving unique functions due to their structural differences.

Hyaline cartilage, composed of collagen fibers, elastic fibers, and an amorphous matrix, is primarily found in the trachea, bronchi, sternal ends of the ribs, and articular surfaces. It plays a crucial role in providing smooth joint movement and cushioning, offering support and flexibility, particularly in areas with lighter load-bearing demands.

Elastic cartilage, similar to hyaline cartilage but with a higher concentration of elastic fibers, provides greater elasticity, allowing it to return to its original shape after deformation. It is primarily found in the auricle, external acoustic meatus, pharyngotympanic tube, epiglottis, and larynx, where flexibility and shape retention are essential.

Fibrocartilage features tightly arranged collagen fibers, providing exceptional resistance to pressure and mechanical stress. It is located in areas such as intervertebral discs, joint cavities, and menisci, where it offers support, cushioning, and protection against high mechanical forces.

## 4 The regenerative capacity of cartilage

Cartilage has limited vascularity, which significantly restricts its regenerative capacity, with chondrocytes being the primary cells involved (Chen et al., 2022a). A key factor in the pathogenesis of osteoarthritis is an imbalance in joint metabolism, where mechanical wear plays a classic role. When catabolic processes surpass anabolic processes, it leads to the degradation of the cartilage matrix. While mechanical wear is often mentioned as a contributing factor, emerging evidence suggests that certain catabolic factors, such as inflammatory mediators and matrix-degrading enzymes, actively contribute to cartilage destruction. However, the mechanisms that halt matrix synthesis and prevent effective regeneration remain largely unclear. In summary, the factors influencing cartilage regeneration include the extracellular matrix, mechanical loading, and genetic regulation, all of which contribute to the disease’s progression.

## 5 Regulatory role of ncRNA in cartilage regeneration

### 5.1 Regulatory role of circRNAs in cartilage regeneration

In recent years, research has begun to elucidate the various roles of circRNAs, including their critical involvement in the occurrence, development, diagnosis, prognosis, and treatment of diseases (Table 2).

**TABLE 2:** List of circRNAs related to OA and cartilage regeneration.

circRNA ID	Expression	Target	Functional role	Reference
circPDE4B	Down	RIC8A and MID1	Promote cell viability and inhibit the catabolic effect	Shen et al. (2021b)
circGNB1	Up	miR-152-3p/RNF219/CA V1 axis	Enhance catabolic factor expression and oxidative stress while suppressing anabolic genes	Liang et al. (2023)
circCDK14	Down	miR-125a-5p/Smad2 axis	Regulate metabolism, inhibit apoptosis, and promote proliferation	Shen et al. (2020)
circRNA2829	Up	miR-4286R + 1/FOXO4	Promote cell proliferation and increase the level of intracellular ALP	Yao et al. (2023)
circSLC7A2	Down	miR-4498/TIMP3 axis	Regulation of cartilage homeostasis and chondrocyte apoptosis involvement	Ni et al. (2021)
circZSWIM6	Up	RPS14-PCK1-AMPK axis	Regulate ECM metabolism and AMPK-associated energy metabolism	Gong et al. (2023)
circHIPK3	Down	miR-124-3p/MYH9 axis	Regulate the expression of key cartilage genes to prevent chondrocyte degradation	Li et al. (2021)
circRSU1	Up	miR-93-5p-MAP3K8 axis	Promote ECM degradation and regulate chondrocyte oxidative stress	Yang et al. (2021)

CircRNAs regulate OA by forming complexes with proteins. Zhou et al. reported the foundational role of circRNA33186 in the development of OA, providing a potential drug target for OA treatment (Zhou et al., 2019b). Shen et al. found a negative correlation between the expression of circPDE4B and the degree of cartilage degeneration, suggesting a potential association between circPDE4B and the occurrence of OA. RIC8A, a guanine nucleotide exchange factor for G protein alpha subunits, was initially discovered in *C. elegans* (Ruisu et al., 2017). Recent studies have revealed that RIC8A plays a role in the progression of OA by modulating the p38 MAPK signaling pathway. The p38 MAPK pathway, along with ERK and JNK pathways, is a key member of the MAPKs family, and its activation is closely associated with OA cartilage damage (Ge et al., 2017; Latourte et al., 2017). Furthermore, circPDE4B has been found to promote the ubiquitination of RIC8A by facilitating the interaction between MID1 and RIC8A, thereby playing a critical role in OA progression (Shen et al., 2021a).

CircRNAs regulate gene expression and, consequently, OA progression through the mechanism of miRNA sponge activity. With an increasing body of evidence emphasizing the connection between miRNAs and the pathogenesis of OA (Cao et al., 2021), miRNAs are considered potential therapeutic targets for OA. Studies have found a significant upregulation of miR-1271 expression in OA, leading to the inhibition of ERG expression (Jones et al., 2009). The expression levels of ERG in the articular cartilage of patients with OA are significantly decreased. CircSERPINE2 acts as a miR-1271 sponge, suppressing chondrocyte apoptosis, promoting ECM metabolism, and inhibiting ECM degradation, thereby slowing down the progression of OA. Overexpression of circSERPINE2 and ERG alleviates ECM degradation. In summary, circSERPINE2 regulates the progression of OA through the circSERPINE2-miR-1271-ERG axis (Shen et al., 2019).

CircRNAs play a regulatory role in OA through miRNA sponge activity in cell engineering. MSCs are considered a promising cellular-based therapy for OA due to their immunomodulatory and multipotent differentiation capabilities (Jones et al., 2009). Adipose-derived MSCs (AMSCs) possess multi-lineage differentiation potential, are more abundantly sourced and easier to isolate than bone marrow-derived MSCs, cause less damage to the donor, and exhibit higher proliferative capacity *in vitro* (Mohamed-Ahmed et al., 2018). AMSCs can also secrete functional cytokines, including molecules inducing cartilage formation (such as TGF-β and FGF), to promote differentiation. *SOX9*, as the master regulator of cartilage, can directly bind to the promoter regions of cartilage-specific genes, such as *Acan* and *Col2*, activating their transcription to promote the synthesis of cartilage matrix and the differentiation of MSCs into chondrocytes. Additionally, it inhibits the expression of *Runx2* and *Col10a1*, preventing the transformation of chondrocytes into hypertrophic phenotypes (Barter et al., 2017). *Acan*, a major component of the cartilage matrix, provides compressive resistance, while *Col2*, the primary collagen in the cartilage matrix, forms a fibrous network that offers tensile strength. Through the balance of synthesis and degradation, as well as interactions with other molecules and growth factors, *Acan* and *Col2* jointly regulate cartilage regeneration. Previous studies have shown that overexpression of miR-145-5p can inhibit cell proliferation and chondrogenic differentiation of human AMSCs (hAMSCs). Silencing miR-145-5p can enhance the osteogenic potential of AMSCs and reduce adipogenesis (Liu et al., 2019b), Moreover, miR-145-5p plays a crucial role in cartilage formation. Jonathan et al. found that miR-145-5p is upregulated during chondrogenic differentiation, possibly regulating MSC cartilage formation by targeting *Sox9*, *Acan*, *Foxo1*, and *Runx3* (Kenyon et al., 2019). During the differentiation of hAMSCs into cartilage, the expression of circATRNL1 is positively correlated with *Sox9*. CircATRNL1 acts as a competitive endogenous RNA (ceRNA) regulating the expression of downstream miR-145-5p and participating in AMSC differentiation into cartilage. CeRNA can act as miRNA sponges, protecting target genes from miRNA suppression. CircATRNL1 may be involved in the cartilage repair process by regulating cartilage differentiation markers such as *Col2*, *Acan*, and *Sox9*. Additionally, circATRNL1 has the potential to inhibit adipogenic differentiation. Through validation, circATRNL1 exerts its function through miRNA sponge activity, with miR-145-5p being a shared target with circATRNL1/SOX9 (Zhu et al., 2021). Therefore, circATRNL1 may promote the chondrogenic differentiation of AMSCs through miR-145-5p sponge activity.

CircRNA regulates OA through miRNA sponge activity within exosomes (Exos). Exos are microvesicles with diameters ranging from 40 to 150 nm, capable of carrying various proteins, lipids, and nucleic acid materials, such as DNA, RNA, mRNA, and ncRNA (Zhang et al., 2021). Exos transport cargo from donor to recipient cells, regulating pathological processes, including OA, chondrogenic differentiation, immune microenvironment, and are produced and secreted by various cell types. Exos derived from MSCs can deliver nucleic acids, proteins, and lipids, providing a favorable microenvironment, enhancing cartilage repair, and slowing down the progression of OA (Toh et al., 2017). Moreover, ceRNA networks play a crucial mechanistic role in various diseases, including OA (Ouyang et al., 2019). Circ0001236, originating from Exos, promotes the chondrogenic potential of MSCs. Its expression is significantly higher in normal cartilage than in OA cartilage. Acting as miR-3677-3p sponge, circ0001236 promotes the expression of Sox9 and Col2, enhancing chondrogenic differentiation. The study found that Exos overexpressing CircRNA0001236 can alleviate ECM degradation, ultimately inhibiting OA progression and promoting cartilage repair (Mao et al., 2021).

**FIGURE 4:**
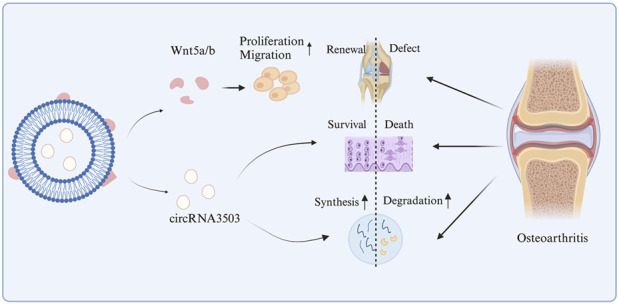
The mechanism of action of circRNA3503 in OA.

In addition, circRNAs can also be combined with biomaterial engineering. In Tao et al.'s study, the binding of sleep-related circRNA3503 with hydrogel was employed to regulate the progression of OA through the Wnt5a/b signaling pathway (Figure 4). Overexpression of circRNA3503 in synovial mesenchymal stem cell-derived EVs (SMSC-sEVs) produced sEVs carrying circRNA3503 (circRNA3503-OE-sEVs). PDLLA-PEG-PDLLA (PLEL) is a triblock copolymer gel that has attracted widespread attention in the research of drug delivery systems and translational medicine due to its injectability, reversibility, and thermosensitivity. Particularly, their ability to self-assemble into core-shell micelles at room temperature and transform into physically cross-linked non-flowing gel structures under physiological conditions is considered an excellent characteristic for nanoparticle/nanodrug delivery (Tang et al., 2021). Furthermore, PLEL as the carrier for sEVs demonstrated excellent performance in sustained release, and PLEL@circRNA3503-OE-sEVs exhibited significant protective potential in OA (Tao S.-C. et al., 2021), confirming the applicability of circRNA in biomaterial engineering.

### 5.2 The regulatory role of miRNA in cartilage regeneration

miRNAs are endogenous, multifunctional ncRNAs with a length of 18–22 nucleotides (Table 3). The synthesis of cartilage matrix is mediated by chondrocytes that constitute the cartilage. Imbalances in ECM synthesis and degradation lead to OA. Pro-inflammatory cytokine interleukin-1β (IL-1β) can induce OA by activating nuclear factor κB (NF-κB), while inducing inducible nitric oxide synthase (iNOS), matrix metalloproteinases (MMPs), and the production of other pro-inflammatory cytokines (Kapoor et al., 2011). miRNAs participate in the progression of OA by mediating chondrocyte survival, inflammatory responses, and extracellular matrix deposition (Fathollahi et al., 2019). As previously reported, silencing miR-146a effectively alleviates joint cartilage damage in an OA mouse model by regulating calcium/calmodulin-dependent kinase II-delta and phosphatase 3, modulating subunits B and β (Zhang X. et al., 2017), MiRNA-93 inhibits apoptosis and inflammation of chondrocytes *in vitro* and *in vivo* by suppressing the toll-like receptor 4/NF-κB signaling pathway (Ding et al., 2018). MiR-29a has a protective effect on synovial deterioration in OA.

**TABLE 3:** List of miRNAs related to OA and cartilage regeneration.

miRNA	Expression	Target	Functional role	Reference
miR-136-5p	Down	ELF3	Inhibit cartilage degeneration in traumatic osteoarthritis	Chen et al. (2020a)
miR-127-3p	Down	CDH11-mediated Wnt/β-catenin pathway	Inhibit CDH11, thereby blocking the Wnt/β-catenin pathway in chondrocytes and reducing the chondrocyte damage in osteoarthritic joints	Dong et al. (2021b)
miR-9-5p	Down	Syndecan-1	Has anti-inflammatory and cartilage protective effects on osteoarthritis	Jin et al. (2020)
miR-29b-5p	Down	TET1	Creat a regenerative microenvironment to mitigate chondrocyte senescence	Zhu et al. (2022)
miR-17	Down	MMP3/13、ADAMTS5、NOS2	Maintain the physiological catabolic and anabolic balance	Zhang et al. (2022)
miR-205-5p	Down	PTEN/AKT axis	Promote the proliferation, migration and anabolism of chondrocytes and inhibit inflammation	Shen et al. (2022)
miR-23a-3p	Up	PTEN、AKT	Promote cartilage regeneration	Hu et al. (2020)
miR-224-5p	Up	TNF	reduce oxidative stress, inhibit apoptosis and promote autophagy	Phillips (2023)

miRNA participates in the progression of OA by mediating chondrocyte survival. Asp-Glu-Ala-Asp (DEAD)-box polypeptide 20, abbreviated as DDX20, is a DEAD-box RNA helicase that is differentially expressed in OA cartilage (Li et al., 2019). DDX20 can activate the NF-κB signaling pathway, downregulating inflammatory responses in chondrocytes. miR-361-5p may serve as a potential marker for early rheumatoid arthritis (Romo-García et al., 2019). Extracellular vesicle miR-361-5p inhibits the NF-κB signaling pathway by downregulating DDX20 in IL-1β-treated chondrocytes, thereby alleviating OA by inhibiting chondrocyte apoptosis (Tao Y. et al., 2021).

miRNA regulates chondrogenesis and cartilage degradation by inhibiting Histone deacetylases (HDACs), affecting the expression of cartilage-specific genes (Lu et al., 2020). Currently, HDACs are categorized into classes I, II, III, and IV based on their function, structure, distribution, and expression patterns. Class I, including HDAC1, 2, 3, and 8, has been reported to play a crucial role in the progression of OA and cartilage formation (Carpio and Westendorf, 2016). Elevated expression of HDAC1 and HDAC2 has been observed in the chondrocytes of OA patients, inhibiting the expression of matrix proteins like Acan and Col2 (Zhang H. et al., 2020). miR-520d-5p is an essential miRNA associated with human MSCs (hMSCs) in chondrogenic differentiation and chondrocyte metabolic activity, and it regulates the process of cartilage generation by inhibiting HDACs. This miRNA plays a crucial role in maintaining cartilage homeostasis and its dysregulation can contribute to the pathogenesis of OA by affecting key signaling pathways and the expression of cartilage-specific genes (Liu et al., 2004).

The role of miRNA in cell engineering for cartilage repair involves the application of MSCs as therapeutic biological carriers in cell therapy. MSCs have gained widespread attention due to their advantages, such as immunomodulation, homing to tumors, easy and rapid isolation, *in vitro* expansion, multilineage differentiation, and the ability to deliver various therapeutic agents (Foo et al., 2021). Synovial MSCs (SMSCs) exhibit significant proliferation and chondrogenic potential, suggesting the possibility of accelerating chondrocyte proliferation to repair cartilage injuries. Loading SMSC-derived EVs with miR-31 has been shown to delay cartilage injury by inhibiting KDM2A, upregulating the E2F1/PTTG1 axis, and downregulating the expression of inflammatory factors IL-1β, IL-6, and TNF-a, thereby preventing the onset of knee joint OA. Among them, KDM2A can reduce the expression of Histone H3 lysine 4 trimethylation (H3K4me3) on the promoter of Secreted Frizzled-Related Protein 2 (SFRP2), inhibiting bone formation and triggering the occurrence of osteoarthritis. Additionally, KDM2A can bind to the transcription factor E2F Transcription Factor 1 (E2F1), inhibiting its transcriptional activity and, consequently, suppressing the progression of OA.

MiRNAs participate in the progression of OA by mediating ECM deposition. Studies have shown that EVs secreted by MSCs can alleviate osteoarthritis by promoting the migration and proliferation of chondrocytes (Zhu et al., 2017). Cadherin-11 (CDH11) has been found to be highly expressed in various fibrotic diseases, including arthritis, and its deficiency prevents inflammatory cell infiltration and cartilage erosion in arthritis mice (Lee et al., 2007). CDH11 overexpression elevates the levels of MMP-13 and ADAMTS-5 factors, weakening the therapeutic effect of EVs (Wang and He, 2018). CDH11 is a downstream target of miR-127-5p, which enriched in EVs, significantly inhibits the levels of these factors and regulates the expression of type II collagen in IL-1β-induced mouse chondrocytes (Zhou et al., 2019c). Numerous key molecules and signaling pathways are involved in the therapeutic capacity of EVs derived from MSCs for bone diseases, including the Wnt/β-catenin pathway (Liu et al., 2019a). EVs-derived miR-127-5p from BM-MSCs inhibits CDH11 in chondrocytes, thereby blocking the activation of the Wnt/β-catenin pathway and protecting chondrocytes from OA damage (Dong et al., 2021a).

MiR-100-5p in the Exos derived from MSCIPFP maintains cartilage homeostasis and protects articular cartilage from injury by inhibiting the mTOR autophagy pathway (Wu et al., 2019a). Exos derived from MSCs are more stable under various physiological conditions than stem cells, to some extent possess immunoprivilege, and have been reported to be effective for cartilage protection (Liu et al., 2017; Zhang et al., 2018). Exos from BMSCs can inhibit cartilage degeneration both *in vivo* and *in vitro* (Mao et al., 2018). Autophagy is a highly conserved degradation process in all eukaryotic cells. Previous studies have shown that autophagy is closely involved in cartilage biology and significantly delays the pathological progression of OA. Cartilage-specific loss of mTOR can protect mice from osteoarthritis by enhancing autophagy activity. Intra-articular injection of rapamycin, an mTOR inhibitor, can activate autophagy and effectively alleviate the pathological phenotype of OA (Caramés et al., 2012). MSCIPFP-Exos can improve the severity of OA *in vivo*, inhibit cell apoptosis, enhance matrix synthesis, and reduce the expression of extracellular matrix degradation factors. Furthermore, MSCIPFP-Exos can significantly enhance the autophagy level of chondrocytes, in part by inhibiting mTOR. MiR-100-5p can reverse the reduced mTOR signaling pathway mediated by MSCIPFP-Exos. *In vivo*, intra-articular injection of inhibitor-miR-100-5p can enhance the articular cartilage protection mediated by MSCIPFP-Exos.

### 5.3 The regulatory role of lncRNA in cartilage regeneration

In recent years, a plethora of long non-coding RNAs (lncRNAs) has been identified in biological processes such as cartilage development, degeneration, and regeneration (Table 4). It is evident that lncRNAs play a crucial role in regulating gene expression and maintaining the phenotype and homeostasis of chondrocytes. Cartilage-related diseases, such as OA and intervertebral disc degeneration (IDD), lead to pain and restricted movement. The primary cause of OA and IDD is the progressive destruction of cartilage (Zhang H. et al., 2017). LncRNA have garnered increasing attention due to their diverse functionality in various tissues. LncRNAs are actually transcribed by RNA polymerase II and include RNA processing signals such as poly(A) tails and 5′caps. Due to the lack of an ORF, lncRNAs were once considered “junk RNA.” However, with the progress of research, it has been discovered that lncRNAs play crucial roles in various biological processes. LncRNAs undergo alternative splicing and a process of intron removal (Tye et al., 2015). With lengths ranging from 200 to 100,000 nucleotides, lncRNAs structurally resemble mRNA transcripts but do not encode protein functions (Hombach and Kretz, 2016). Based on their relative positions to gene loci, lncRNAs can be classified into five categories: sense, antisense, bidirectional, intronic, and intergenic (Li and Wang, 2015). Compared to protein-coding genes, lncRNAs exhibit stronger species specificity and lower conservation. Past research on lncRNAs is summarized in Table 4.

**TABLE 4:** List of LncRNAs related to OA and cartilage regeneration.

LncRNA	Expression	Target	Functional role	Reference
MIR22HG	Up	miR-9-3p/ADAMTS5	Overexpression of matrix metalloproteinase	Long et al. (2021)
PVT1	Up	miR-140	Promote ECM degradation in chondrocytes	Yao et al. (2022)
ARFRP1	Up	miR-15a-5p/TLR4	Induce the injury of chondrocytes	Zhang et al. (2020a)
ZFHX2	Up	KLF4	Promote chondrocyte DNA repair	Ni et al. (2023)
02035	Up	RUNX2	Stabilize RUNX2 protein and induce hypertrophic changes	Yoon et al. (2023)
NEAT1	Up	miR-193a-3p/SOX5	Promote ECM degradation in chondrocytes	Liu et al. (2020)
H19	Down	miR-483-5p/Dusp5	Inhibit cartilage degeneration	Wang et al. (2020)
KLF3-AS1	Down	PI3K/Akt/mTOR	Repress autophagy and apoptosis of chondrocytes	Wen et al. (2022)
CRNDE	Down	SIRT1/SOX9	Regulates BMSC chondrogenic differentiation	Shi et al. (2021)
Malat-1	Down	MMP-13, IL-6, and Caspase-3	Promote chondrocyte proliferation and migration, suppressed apoptosis	Pan et al. (2021)
AC006064.4–201	Down	PTBP1	Downregulate the translation of CDKN1B protein	Shen et al. (2023)

Recent studies have indicated that lncRNAs can regulate coding mRNA by competitively binding to miRNAs; this class of lncRNAs is referred to as ceRNA (Salmena et al., 2011). ADAMTS9-AS2 belongs to the antisense lncRNA category, playing a crucial role in the regulation of gene expression and genomic integrity. Similar to BACE1-AS, it originates from the antisense orientation of the BACE1 gene and can enhance BACE1 expression through the formation of RNA double-stranded nucleotides. Unlike ADAMTS9-AS2, there is no impact on the expression of the ADAMTS9 gene. Instead, ADAMTS9-AS2 functions as a ceRNA, targeting miR-942-5p. LncRNA ADAMTS9-AS2 promotes chondrogenesis both *in vitro* and *in vivo*. Implanting hMSCs overexpressing ADAMTS9-AS2 into bone cartilage defects significantly improves cartilage repair. SCRG1 is a transcript initially identified in mouse infectious spongiform encephalopathy through the identification of genes associated with observed neurodegenerative changes. Research indicates that lncRNA ADAMTS9-AS2 regulates the transcription factor SCRG1 as a sponge for miR-942-5p, thereby promoting hMSC differentiation into cartilage both *in vitro* and *in vivo* (Huang M.-J. et al., 2019). Research has demonstrated that lncRNA SNHG1 effectively reduces reactive oxygen species (ROS) levels, enhances mitochondrial membrane potential, and promotes ATP production, thereby regulating mitochondrial function and facilitating cartilage repair (Liu et al., 2024).

Furthermore, lncRNAs can mediate the balance of ECM in OA chondrocytes. For instance, knocking down lncRNA-CIR accelerates chondrocyte apoptosis, inducing cartilage degradation (Liu et al., 2014). LncRNA CASC2 is upregulated in OA. Overexpressing CASC2 inhibits chondrocyte proliferation and promotes chondrocyte apoptosis by upregulating IL-17 (Huang T. et al., 2019). Overexpression of lncRNA FOXD2-AS1 promotes chondrocyte proliferation in OA by sponging miR-27a-3p (Wang et al., 2019). Previous research suggests that lncRNA SNHG15 can mediate various human cancers by sequestering multiple miRNAs (Ye et al., 2019). A recent study reported downregulation of SNHG15 in OA cartilage tissue and IL-1β-induced chondrocytes (Zhang X. et al., 2020). Inhibiting SNHG15 reduces chondrocyte apoptosis and ECM degradation, improving joint cartilage injury, indicating the involvement of SNHG15 in OA progression. KLF4 is a zinc finger transcription factor involved in various biological processes such as cell differentiation and tissue formation (Chen et al., 2019). KLF4 has been confirmed to be downregulated in human OA cartilage tissue. miR-7 is underexpressed in OA cartilage tissue and IL-1β-induced chondrocytes. KLF4 is a target of miR-7, and its low expression inhibits the release of inflammatory cytokines and cell apoptosis in IL-1β-induced chondrocytes (Zhou X. et al., 2019). β-catenin has been identified as a functional downstream effector of KLF4. Overexpression of KLF4 can suppress β-catenin expression. LncRNA SNHG15 can mitigate the progression of OA by regulating ECM homeostasis through the modulation of the miR-7/KLF4/β-catenin axis, thereby influencing OA (Chen Y. et al., 2020).

### 5.4 The regulatory role of siRNA in cartilage regeneration

RNA interference (RNAi) has been considered a promising approach for targeted therapy. In targeted therapy, defective genes can be edited by delivering complementary siRNA in the post-transcriptional stage. Successful editing of specific genes through delivered siRNA may slow down or halt the progression of OA without causing side effects associated with chemical inhibitors. However, the delivery of siRNA to cartilage remains a challenging target due to the avascular and highly dense nature of cartilage tissue, leading to low permeability.

**FIGURE 5:**
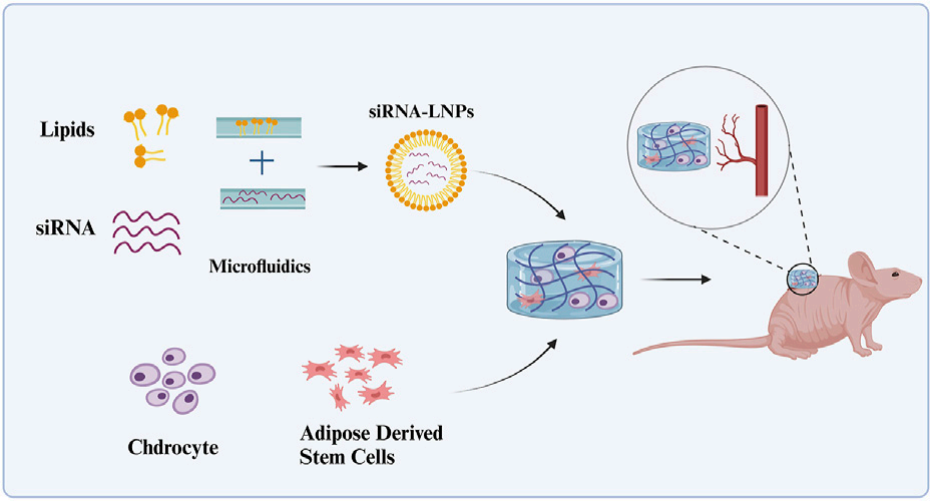
The VEGFα siRNA encapsulated in LNPs promotes cartilage formation by inhibiting angiogenesis.

In addition to stem cell strategies, another challenge currently faced in the field of cartilage tissue engineering is how to generate cartilage *in situ* in the body and stabilize it. Internal inflammation and vascular environments may have negative effects on cartilage *in situ* regeneration (Ji et al., 2021). Vascular invasion promotes ossification and tissue mineralization, inducing hypertrophy of chondrocytes. Vascular endothelial growth factor alpha (VEGFα) is one of the key factors in angiogenesis, guiding and supporting the process of vascular infiltration. Chen et al. proposed that promoting cartilage tissue regeneration can be achieved by inhibiting vascular infiltration through blocking VEGFα (Chen et al., 2022b). To block VEGFα, Chen et al. utilized RNA interference (RNAi). Transfecting siRNA into recipient cells is a challenge due to the high hydrophilicity, high molecular weight, and anionic charge characteristics of siRNA. Additionally, siRNA is rapidly degraded and inactivated in the body. Silk fibroin nanoparticles, sodium alginate nanoparticles, polypyrrole nanoparticles, and LNPs are widely employed in drug delivery due to their high surface area-to-volume ratio, stability, and modifiable size (Steinman et al., 2020). Among these, LNPs exhibit characteristics such as low toxicity, immunogenicity, and a low risk of mutagenicity, leading to their FDA approval for siRNA delivery (Yonezawa et al., 2020). Additionally, employing a dual delivery strategy by encapsulating LNPs in natural or synthetic polymers enables more controlled and sustained drug release, enhancing targeting and stability while reducing side effects. GelMA possesses excellent biocompatibility, suitable biodegradability, and mechanical strength (Yue et al., 2015). VEGFα siRNA-LNPs prepared using microfluidic technology exhibit efficient transfection capability and good intracellular escape properties. Embedding VEGFα siRNA-LNPs into methacrylated gelatin (GelMA) hydrogels constructs an engineered cartilage scaffold, providing additional control for localized release and effective delivery of VEGFα siRNA. After constructing the GelMA/cells + VEGFα siRNA-LNPs complex, it is subcutaneously implanted into nude mice, demonstrating its ability to inhibit neovascular infiltration in developing cartilage while achieving stable regeneration of transparent cartilage (Figure 5).

In OA, chondrocytes undergo phenotypic changes and aging, limiting cartilage regeneration and accelerating disease progression. p16INK4a is a senescence biomarker that can induce aging by halting the cell cycle. Park et al. encapsulated p16INK4a siRNA in poly (lactic-co-glycolic acid) (PLGA) nanoparticles and characterized them. Molecular analysis and behavioral tests were performed on the OA model using partial medial meniscectomy (pMMx). The results showed that p16INK4a siRNA-loaded PLGA nanoparticles (p16 si-NP) reduced levels of TNF-α, IL-1β, and IL-6, especially MMP13 in fibroblast-like synoviocytes (FLSs) and chondrocytes, contributing to the recovery of osteoarthritic cartilage and pain relief (Park et al., 2022).

### 5.5 Disscussion

NcRNAs are key regulatory factors in cartilage regeneration, offering significant therapeutic potential for cartilage injuries and degenerative diseases such as OA. Previous *in vitro* and *in vivo* studies have demonstrated the critical roles of ncRNAs in chondrocyte proliferation, differentiation, and ECM metabolism. *In vitro* studies, conducted in controlled environments, have elucidated specific mechanisms, such as the roles of miR-145-5p and circATRNL1 in chondrogenic differentiation by targeting *Sox9* and *Col2* (Zhu et al., 2021; Shen et al., 2021b). *In vivo* studies, on the other hand, provide a more physiologically relevant context. For instance, lncRNA ADAMTS9-AS2 and circRNA0001236 have been shown to promote cartilage repair by modulating chondrocyte differentiation and ECM homeostasis in rat and mouse models (Huang M.-J. et al., 2019; Mao et al., 2021).

The clinical translation of ncRNA-based therapies faces challenges, particularly in developing effective delivery systems. NcRNAs are unstable and prone to degradation, while the avascular and dense nature of cartilage complicates their delivery. Various strategies, such as LNPs, exosomes, and hydrogels, have been explored. LNPs have shown promise in delivering siRNA to chondrocytes, as demonstrated by Chen et al. (2022b), who used VEGFα siRNA-LNPs to inhibit angiogenesis and promote cartilage regeneration. Exosomes derived from MSCs have been utilized to deliver ncRNAs, such as miR-100-5p, which protects cartilage by inhibiting the mTOR pathway (Wu et al., 2019b). Hydrogels, particularly thermosensitive ones, enable sustained release and localized delivery to cartilage defects (Tao S.-C. et al., 2021). However, these delivery systems still face challenges, including the immunogenicity of certain carriers and potential off-target effects of ncRNAs. Future research could focus on developing more targeted and biocompatible delivery systems, such as exosome-based carriers, and integrating ncRNA therapies with tissue engineering approaches, such as MSC-seeded scaffolds, to enhance cartilage repair.

In summary, ncRNAs hold great promise for cartilage regeneration, but their clinical translation requires overcoming challenges related to delivery, stability, and species specificity. A multidisciplinary approach combining advanced delivery systems, tissue engineering, and a deeper understanding of ncRNA biology is essential for developing effective therapies, ultimately improving outcomes for patients with cartilage injuries and degenerative joint diseases.

## 6 Conclusion

This review highlights the therapeutic strategies for cartilage regeneration, focusing on current treatments, tissue engineering approaches, and advancements in regenerative medicine. NcRNAs, including miRNAs, lncRNAs, circRNAs, and siRNAs, are increasingly recognized for their critical roles in regulating chondrocyte proliferation, differentiation, and ECM synthesis. Their potential as therapeutic tools for cartilage regeneration is substantial, especially in addressing conditions such as OA, where self-repair capabilities are limited.

Recent advancements have shown promising results with ncRNA-based therapies, including the use of exosomes derived from MSCs to deliver ncRNAs like miR-100-5p and circRNA0001236, which have demonstrated efficacy in cartilage repair and the inhibition of OA progression. Furthermore, the combination of ncRNAs with biomaterials, such as hydrogels and lipid nanoparticles, has enhanced their stability and targeted delivery, improving their therapeutic potential.

Looking forward, optimizing delivery systems for ncRNAs, improving their specificity, and minimizing off-target effects will be essential for the clinical success of ncRNA-based therapies. The integration of gene editing technologies such as CRISPR/Cas9 offers exciting possibilities for more precise regulation of cartilage-related genes. Personalized therapies, tailored to individual genetic and epigenetic profiles, hold significant promise for improving treatment outcomes.

In summary, ncRNAs represent a versatile and powerful tool in cartilage regeneration. Continued research into their regulatory mechanisms and therapeutic applications is essential for developing more effective and targeted approaches to treat cartilage injuries and degenerative diseases, ultimately enhancing the quality of life for patients worldwide.
